# A Low-Cost, Open Source Monitoring System for Collecting High Temporal Resolution Water Use Data on Magnetically Driven Residential Water Meters

**DOI:** 10.3390/s20133655

**Published:** 2020-06-29

**Authors:** Camilo J. Bastidas Pacheco, Jeffery S. Horsburgh, Robb J. Tracy

**Affiliations:** 1 Department of Civil and Environmental Engineering, Utah State University, Logan, UT 84322-8200, USA; jeff.horsburgh@usu.edu; 2 Utah Water Research Laboratory, Utah State University, Logan, UT 84322-8200, USA; joshuatracy64@gmail.com

**Keywords:** smart metering, residential water use, high resolution data, Arduino, open source, water end uses, datalogging

## Abstract

We present a low-cost (≈$150) monitoring system for collecting high temporal resolution residential water use data without disrupting the operation of commonly available water meters. This system was designed for installation on top of analog, magnetically driven, positive displacement, residential water meters and can collect data at a variable time resolution interval. The system couples an Arduino Pro microcontroller board, a datalogging shield customized for this specific application, and a magnetometer sensor. The system was developed and calibrated at the Utah Water Research Laboratory and was deployed for testing on five single family residences in Logan and Providence, Utah, for a period of over 1 month. Battery life for the device was estimated to be over 5 weeks with continuous data collection at a 4 s time interval. Data collected using this system, under ideal installation conditions, was within 2% of the volume recorded by the register of the meter on which they were installed. Results from field deployments are presented to demonstrate the accuracy, functionality, and applicability of the system. Results indicate that the device is capable of collecting data at a temporal resolution sufficient for identifying individual water use events and analyzing water use at coarser temporal resolutions. This system is of special interest for water end use studies, future projections of residential water use, water infrastructure design, and for advancing our understanding of water use timing and behavior. The system’s hardware design and software are open source, are available for potential reuse, and can be customized for specific research needs.

## 1. Introduction

The vast majority of water meters used by water supply utilities today for quantifying residential water consumption are analog, magnetically driven, positive displacement meters. These meters use a nutating disc or a similar mechanism and measure water flow using the positive displacement principle. Water flows into a chamber in the meter causing the disk to nutate, and each nutation represents a fixed volume of water. The count of nutations is registered by the meters using a magnetically driven register. Measurements made by these meters are typically within 0.25−0.5% of the actual value [1]. Although these meters are highly accurate and have been used effectively for decades to quantify residential water use for billing purposes, they were designed to be read only periodically, typically monthly or quarterly. Since monthly resolution data provide little information about the distribution of use across end uses (e.g., toilets, showers, faucets, etc.) and the timing of use both within and outside a home, data at a higher temporal resolution must be collected to effectively identify and understand water use behavior. Smart meters have potential to meet this need while supporting automated billing processes. The term “smart meter” can be ambiguous [1]. In this article, the term is related to devices capable of collecting high temporal resolution data (i.e., high sampling frequency) that can be integrated in efficient systems for data management [2]. However, replacing traditional meters with smart meters can be expensive, labor intensive, and disruptive. In consequence, collecting high temporal resolution water use data can be cost prohibitive for many utilities and researchers. Yet, doing so enables new opportunities for quantifying water use behavior at high temporal resolution [3,4,5].

Given that most water meters installed and operating today are not capable of recording high temporal resolution data, many past research studies requiring this type of data have relied on proprietary data collection devices and software that require operation by and input from trained analysts [2]. These data logging devices are installed on top of existing meters using different types of sensors to collect high temporal resolution water use data and, thus, to add smart metering capabilities. Collected data are then downloaded and processed to identify and disaggregate end uses of water. However, the associated costs can be prohibitive for many researchers and water utilities. For example, DeOreo et al. [6,7] used Meter-Master flow recorders [8] to collect 10 s resolution data for hundreds of households. This proprietary device is installed on positive displacement, magnetically driven meters and can collect high temporal resolution data, but a single unit can cost over $2000 USD. Other authors have developed and tested different sensors and data-recording devices to identify when a fixture is used within the house [9,10,11,12]. With these technologies, disaggregation of end uses requires the existence of a smart meter capable of high temporal resolution data collection. These devices can identify when, and in some cases where, a fixture is being used, but rely on postprocessing of the smart meter data to estimate volumes, flow rates, and other characteristics of the events. Some devices used in other studies to collect high temporal resolution data [9] are not available today because they have been sold to private partners [13].

There are commercially available smart meters that can collect data at the minute resolution [14], but this may be too coarse for some applications (e.g., identifying individual end use events) because some events have durations that last only seconds. Other devices are entering the market that are designed to collect data at higher temporal resolutions for the purpose of detecting leaks and providing information to water consumers [13,15]. These devices are proprietary (i.e., they are produced by commercial companies for sale, cannot be modified, and source code is not open), they are not interoperable, and they generally do not provide access to the raw data they collect, opting instead to provide water consumers with summary information designed to inform them about their water consumption. Although promising for consumer applications, these devices are not well suited for research data collection. Thus, an openly available and affordable data collection device could solve one of the existing limitations for conducting research using high temporal resolution water use data.

Over the past several years, there has been a general reduction in prices of sensors and dataloggers; however, cost continues to be an important limitation for scientific research [16,17]. More recently, open-source electronics hardware, specifically Arduino, has been identified as a viable alternative for expensive, commercial instrumentation in scientific research [18]. Arduino is an open-source electronics prototyping platform that consists of both microcontroller hardware and the Arduino software for programming them [19]. In the field of water resources, Arduinos have been used in multiple applications that range from monitoring water quality in streams [20], promoting water conservation [21], operating irrigation systems [22,23], and many other applications [24,25]. One of the strengths of the Arduino is the Integrated Development Environment (IDE) that includes extensive code libraries for developing measurement and control systems [26]. Arduinos are highly configurable computing devices that have expanded the development of customized applications in multiple fields. They can be transformed into autonomous systems, installed in tiny spaces, used in remote field locations, and they can be deployed without peripheral devices like monitors and keyboards [27]. The availability of Arduino-compatible development boards has helped to create a new variety of inexpensive, open-source hardware for data logging applications [26].

In this paper, we describe an open-source datalogger that uses an Arduino microcontroller board in combination with other commonly available hardware components to measure and record high temporal resolution water use data on analog, magnetically driven, positive displacement meters. Developed as part of a larger effort aimed at developing Cyberinfrastructure for Intelligent Water Supply (CIWS), the CIWS datalogger can be used with existing meters without affecting their functioning or their normal data collection activities, either manual or wireless. Thus, adding a CIWS datalogger to an existing, analog meter effectively transforms it into a smart meter capable of recording data at any temporal resolution required for a particular study. The hardware and software of the CIWS datalogger are open source, and they can be modified to fit specific research needs. The CIWS datalogger software uses existing Arduino code libraries, and new libraries were also developed for specific functions. The system presented is a low-cost alternative for collecting high temporal resolution residential water usage data. The main characteristics we sought to meet in the design of this system included: ease of assembly, autonomous operation for approximately 6 weeks while recording data at high temporal resolution (<5 s), low purchase and assembly cost, flexibility for customization, accuracy of measurements, and building from an open hardware and software platform.

This article is organized as follows: Section 2 describes the CIWS datalogger, its functioning principle, hardware design, software, and user interface. Section 3 presents the procedures we used to test and calibrate the device in a laboratory setting using multiple meters from different manufacturers along with calibration results. Section 4 discusses the results of a field deployment campaign we used to test the data-collection capabilities and functioning of the device under normal operating conditions. The results of analyses conducted on the data collected are included in this section. Section 5 presents final discussion points, areas for improvement, and future work. The Hardware, Firmware, and Data Availability section at the end of this article provides links to directories where readers can find: (a) hardware designs along with instructions for performing all of the hardware modifications described and a diagram of connections, (b) printed circuit board (PCB) designs and all information required to manufacture them, and (c) firmware code along with more detailed documentation about the organization and functioning of firmware, and (d) data and scripts to reproduce calculations presented here.

## 2. System Description

The CIWS datalogger was designed to operate on top of existing, magnetically driven residential meters of common sizes (e.g., 1 in, 3/4 in, and 5/8 in). In this paper, the meter sizes are described in inches to match manufacturer specifications for how these meters are sold in the United States. The meters used to calibrate and test the CIWS datalogger were manufactured by Neptune and Master Meter and were designed to operate at different flow rates depending on their size. For 3/4 and 5/8 in meters, the manufacturers report accuracy information for flow rates between 0.1 and 20 gallons per minute (GPM). For 1 in meters, the accuracy is reported between 0.35 and 50 GPM. We designed the CIWS datalogger to meet the following specifications: (a) operation on top of existing meters without requiring replacement of the meter and without affecting the function of the existing meter; (b) autonomous operation for longer than 20 days; (c) versatility to work with different meter brands and sizes without requiring in situ calibration; (d) sufficient accuracy and temporal resolution to allow the identification and classification of end uses of water in a residential home; (e) simplicity of use with an easily operable user interface; and (f) output data in an accessible format that is platform and software independent.

### 2.1. Principle of Functioning

Many existing residential water meters use a nutating disc or other similar device to measure water flow using the positive displacement principle. Water flows into a measurement chamber in the meter that obstructs the flow, and a nutating or rotating mechanism allows the passage of a fixed volume of water. Actuation of the chamber’s fixed volume, or displacement, as a nutation or revolution of the measurement element represents passage of a fixed volume of water. The rate of revolution or nutation is proportional to the flow rate. The count of revolutions or nutations is recorded using a magnetically driven register. A magnet inside the register is paired with a spinning magnet inside of the meter’s sealed housing. As water flowing through the meter causes the magnet inside the meter housing to rotate, the paired magnet inside the register also rotates. These rotations are counted by the meter’s register to record the count of pulses, which determines the flow volume and rate. The registers used with most meters provide only volume information, while some registers may also provide a flow rate. However, registers do not provide access to the magnetic pulse information from which the volume and flow rate are derived, and meter manufacturers do not typically publish the pulse resolution (volume of water per pulse) of their meters.

The magnets in the meter and register create oscillations in the magnetic field surrounding the meter as they rotate, generating peaks that can be measured with each revolution. Just like the meter’s register, the CIWS datalogger counts the number of times the magnet inside the meter rotates. The main difference is that it detects the rotations using a magnetometer sensor mounted on the outside of the register. The magnetometer measures changes in the magnetic field as the magnet inside the meter rotates, and the datalogger then counts the peaks that occur in the magnetic field without modifying or affecting the regular function of the meter. The datalogger operates via a firmware code that has two main functions. First, it detects and sums the number of magnetic pulses (peaks) that occur during a time step. Second, it logs this value along with the corresponding date and time. The recording interval is configurable to allow for adequate identification and separation of short-duration water use events. Detailed descriptions of the hardware and software are provided in the sections that follow.

**Figure 1:**
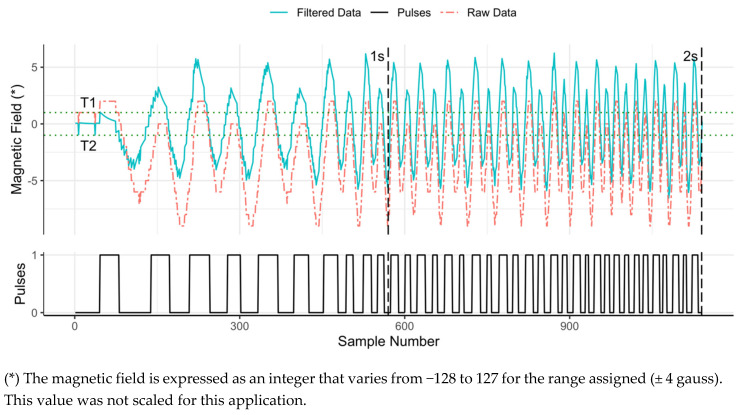
Pulse detection process. The red dashed line represents the raw data collected by the magnetometer. The blue line represents the filtered signal (using Equation (2)), and the black line is the output of the digital Schmitt Trigger, the pulses that are counted and logged by the system. T1 and T2, the green dotted lines at 1 and −1, are the two thresholds used in the Schmitt Trigger function. At the sampling resolution selected, the CIWS datalogger collects 560−570 readings every second. The 1 and 2 s vertical dashed lines are superimposed on the plot at the location of the sample number that corresponds to those time steps.

The magnetometer raw output is a linearly scalable integer between −128 and 127. For counting peaks in the magnetic field, scaling the raw signal does not provide any additional information as the exact value of the magnetic field is not of interest, but only the number of peaks. The raw magnetic field was observed in multiple experiments to characterize it and define an algorithm that could potentially count peaks from any magnetically driven meter, independent of the position of the magnetometer sensor relative to the meter register. In these experiments, the magnetic field was sampled at 570 Hz, and some characteristics of the signal were observed. First, the magnetic field signal is weak, being contained by a small percentage of the ± 4 gauss range, which is the smallest range possible with the sensor selected. Second, peaks are closer to each other, in time, when the flow rate is higher, meaning the frequency of the signal is variable and proportional to the flow rate, which depends on the meter size, the pressure in the pipe, and the fixture through which water is being used. The maximum frequencies observed in our laboratory setting were below 50 Hz, although higher values may be possible in other settings. Third, the range and the average amplitude of the signal are different for every brand and model of meter and are also dependent on the position of the magnetometer relative to the meter. We observed values of the linearly scalable integer between −25 and 3 during laboratory experiments, but different values are possible depending on how the sensor is installed on the meter. Fourth, the value output by the magnetometer sensor can remain constant at any value within the observed range when the magnet stops spinning. Fifth, the signal is noisy. In the upper panel of Figure 1, the red dashed line presents the raw output of the magnetometer during a 2 s data recording interval. When starting the data collection, the valve controlling water flow through the meter was closed. It was then opened, and the variation in the signal can be observed as water flow was increased.

The approach designed to count peaks uses two thresholds, T1 and T2 (Figure 1). A pulse is counted when the signal goes above T1 and then sequentially below T2. A single threshold approach is not sufficient given that the magnetic field can remain constant at any value within its range and the noise in the signal that can cause oscillations above and below a single threshold that do not represent true pulses. Additionally, observing local maximums within a fixed number of values is not possible due to the changing period in the signal as flowrates change. Since the average amplitude of the signal is not constant, these two thresholds would need to be calibrated for every installation (i.e., every type of meter and sensor location), which would limit the generalization of the application. To address this, an infinite impulse response (IIR) filter was added to process the magnetometer output to produce a signal with constant mean amplitude independent of the meter type and installation location of the magnetometer sensor. Digital filters, including IIR filters and finite impulse response (FIR) filters [28], are fundamental in processing signals to remove their unwanted parts. The main difference between them is that IIR filters are recursive and use feedback from the output in the filter structure, whereas FIR does not [29]. IIR filters have a higher computational economy because they require less memory and fewer arithmetic operations than FIR [30]. This makes them better suited for this application, which requires running the algorithm on the microcontroller in real time. Recording the raw data and processing it later in a centralized facility would require larger computational power given the volumes of data that are generated since the magnetic field is sampled at a 560−570 Hz rate. However, IIR filters need to be designed with extra care because they can become unstable [30].

IIR filters are typically expressed as a difference equation, which calculates a sample output at a time *n* based on past outputs and present and past inputs. The order of a difference equation is defined by the number of past samples it uses [31]. A basic, first order, difference equation form is presented in Equation (1):

(1) $$y_{n}={a\;*\;y}_{n-1}+b_{1}{\;*\;x}_{n}-b_{2}x_{n-1}$$

where, $y_{n}$ is the output filtered signal for the current time step, *n*; a is typically known as the feedback coefficient; $y_{n-1}$ is the output filtered signal for the previous time step, *n* − 1; *b*_1_ and *b*_2_ are the feedforward coefficients; and $x_{n}$ and $x_{n-1}$ are the inputs (raw signal) for the current and previous time steps, respectively [31]. If a is not zero, Equation (1) defines an IIR filter. The feedback and feedforward coefficients are predefined for classical filters, such as Butterworth, Chebyshev, or other designs [30]. For our application, the purpose of the filter was simply to output a signal with a constant average amplitude, rather than to pass or reject specified frequencies, as these filters are typically designed [29], which makes the problem simpler. The feedback and feedforward coefficients are typically calibrated to obtain the response desired. For our application, *b*_1_ and *b*_2_ were set to 1, and *a* was set to 0.95, resulting in Equation (2):

(2) $$y_{n}=0.95{\;*\;y}_{n-1}+x_{n\;}-x_{n-1}$$

**Figure 2:**
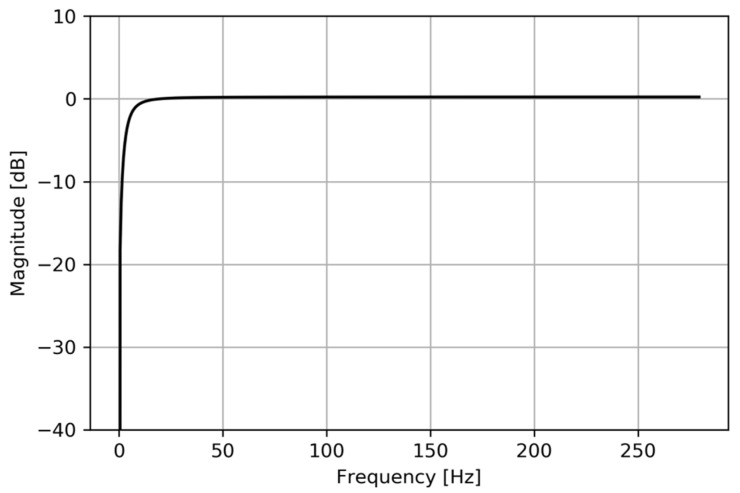
Frequency response of the infinite impulse response (IIR) filter designed for this application.

An IIR filter is stable if its response to an impulse approaches zero as *n* goes to infinity. With the parameters selected, $y_{n}$ will decay gradually if the input is an impulse (i.e., a one followed by zeros). Then, Equation (2) represents a filter that produces a signal with constant mean amplitude equal to zero, that is independent of the meter type and installation location of the magnetometer, and that reduces noise while maintaining the shape of the input signal. These parameters were selected and tested to be valid for this application, but there are infinite configurations of *a*, *b*_1_, and *b*_2_ that would result in a stable filter that would satisfy our requirements (i.e., the output signal must have a constant average amplitude and maintain the shape of the input signal). For example, keeping the same values of *b*_1_ and *b*_2_, any value for *a* larger than 0.95 but less than 1 will meet all requirements while maintaining stability. If we gradually select values for *a* less than 0.95, we will reach a point where the output signal will have fewer peaks than the original signal. Any value of *a* larger than this number and less than 1 will meet our requirements. Having an output signal with the same shape as the input assures that the pulses counted in the output signal also exist in the input signal measured by the magnetometer. The existence of noise does not interfere with the two threshold approach to count pulses because the magnitude of the noise is much smaller than the magnitude of the overall signal. Therefore, finding the values of *a*, *b*_1_, and *b*_2_ that remove the most noise while maintaining the shape is not of interest, but could be easily done, if needed, in laboratory testing. The parameters selected are a valid and simple solution for our application. Figure 2 shows the frequency response of the filter designed. Signals with a frequency near 0 Hz are attenuated, whereas signals with higher frequencies pass through without any attenuation. Since these very low frequencies, especially any 0 Hz component, are so heavily attenuated, the signal loses its constant offset and becomes centered around zero. Lyons [32] provides a more detailed discussion around this type of filter. This filter operates adequately for the frequency ranges described above. Since this is a discrete filter, any frequencies above half of the sampling frequency will alias to a lower frequency signal, and the resulting data will be faulty.

Having a signal with a constant mean amplitude, zero in this case, allows us to define fixed values for the thresholds—in our case, T1 = 1 and T2 = −1. These values were selected based on observations made of the raw signal from multiple water meters and have been proved valid in the field. Similar to the parameters in the filter, there are multiple options for T1 and T2 that would provide a valid solution. The process of counting pulses using two thresholds can be referred to as a digital Schmitt Trigger. The main function of the Schmitt Trigger is to convert the filtered signal, (blue, solid line, upper panel, Figure 1) into a clean, square wave (black, solid line, lower panel, Figure 1) from which pulses can be easily counted. The CIWS datalogger keeps track of time using a real-time clock (RTC) incorporated in the datalogging shield and logs time and the count of pulses in regular, configurable, time step intervals.

### 2.2. Sample Output

**Figure 3:**
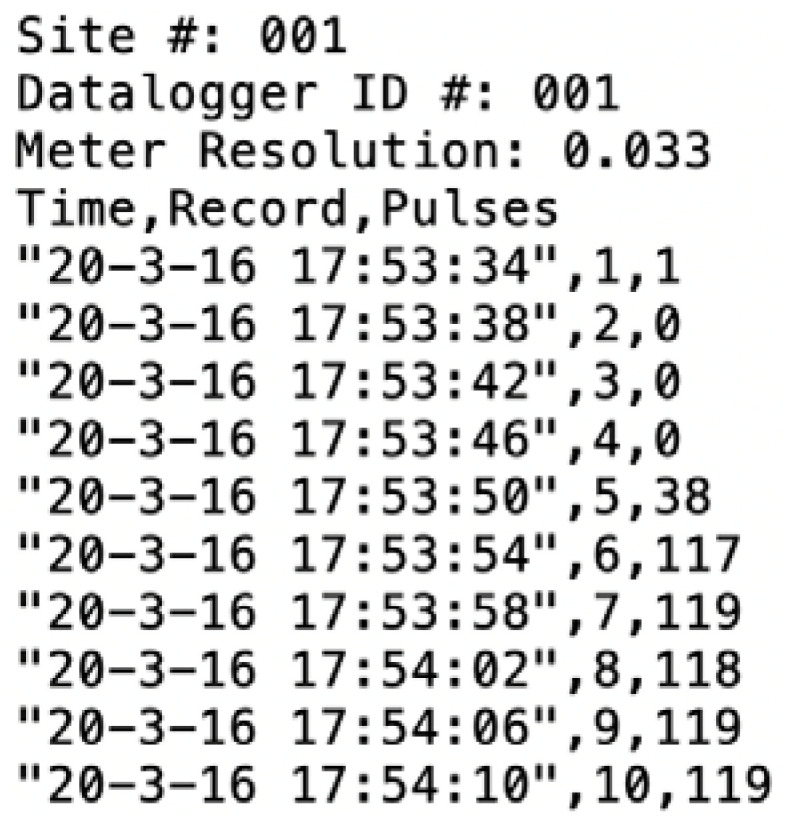
Sample output from the Cyberinfrastructure for Intelligent Water Supply (CIWS) datalogger collecting data with a 4 s time interval. The data is stored in a comma separated values (CSV) file, which is easily operable in multiple platforms and software.

The CIWS datalogger outputs a comma separated values (CSV) file including a 3 line header with information about: (1) Site #, a 3 digit numerical ID used to keep track of where the logger is installed; (2) Datalogger ID #, a 3 digit numerical ID used to identify a datalogger, and; (3) Meter Resolution, a numeric value with 3 decimal places indicating the pulse resolution of the meter (gallons per pulse to match the meter’s register units) where the logger is installed. The meter resolution is used for displaying volumes in the user interface. The logger registers data by keeping track of 3 variables: (1) Time, a datetime value including the date and time in format “Year-Month-Day Hour:Minute:Second”; (2) Record, a numerical ID used to keep track of the number of values logged; and (3) Pulses, an integer indicating the number of pulses registered in a time interval. The datetime string format was chosen to be consistent with the International Standards Organization (ISO) 8601 standard for the representation of dates and times to make it easier to work with across computer operating systems, database programs, and programming languages. Figure 3 shows an example of a CSV file obtained from the CIWS datalogger. In this example, the site and datalogger ID are 001 and meter pulse resolution is set to 0.033. Only the first 10 records are presented.

### 2.3. Hardware

The CIWS datalogger main components are a LIS3MDL digital output magnetic sensor [33], an Arduino Pro microcontroller board [34], and a custom sensor interface board assembled on an Adafruit datalogging shield [35].

#### 2.3.1. Magnetometer Sensor

The LIS3MDL is an ultralow-power, three-axis magnetometer that can operate at different gauss scales (±4, ±8, and ±16 gauss). In this system, we use only one (the x) axis available on the sensor as the y and z axes do not provide additional information for this application. The sensor is configured to operate in the ± 4 gauss scale as the magnetic signal from the water meter is weak enough to be fully captured within this range. The highest flow rate we have observed at residential homes is ~80 liters per minute (LPM), for meters of smaller sizes (3/4 and 5/8 in). With water flowing at that flow rate, we will observe ~40 pulses per second. Using the definition of pulses explained in the previous section, this means the signal will have ~40 positive peaks and the same number of negative peaks when water is flowing at this rate. Higher flow rates are possible. The Nyquist–Shannon sampling theorem establishes that if we want to properly characterize a signal, we must sample it with at least twice the input signal frequency [36]. The frequency of the magnetic signal from the magnetometer changes with the flow rate. In consequence, the sensor must continuously sample at a high rate in order to capture these changes.

The sensor has four system modes: continuous-measurement mode, single-measurement mode, and two idle modes, along with four operating modes, i.e., low-power, medium performance, high-performance, and ultrahigh-performance. Multiple output data rate options are available by choosing an appropriate operating mode, ranging from 0.65 to 1000 Hz [33]. The magnetic data are sent to different registers. For the sensor’s x axis, two registers are used, OUT_X_H and OUT_X_L. These contain the most significant and least significant part of the magnetic signal on the x axis, respectively [33]. A FAST_READ option is also available to accelerate the process of reading data from the sensor. By selecting this option, only the OUT_X_H register data is sent [33]. The sampling frequency of the magnetic signal using the LIS3MDL magnetometer is then a function of the combination of the options selected for each one of these parameters.

In the CIWS datalogger, the sensor system mode is set to the continuous-measurement mode, the operating mode is set to medium performance, and the FAST_READ option is active, which results is a sampling frequency of approximately 560−570 Hz. Other configuration options to sample at 165 and 300 Hz are available. These configuration options were tested in the laboratory for the range of flow rates we observed at residential homes using different meters. Results showed that sampling at 165 and 300 Hz can capture the signal as accurately as sampling at the faster rate. Power consumption differences between these configurations were not estimated, but the slower sampling rates may consume less power and result in longer potential deployment times. The 570 Hz configuration settings were selected for field deployment as a higher sampling frequency results in a better characterization of the signal. This frequency has proved to be sufficient to accurately capture the pulses associated with water flowing at the maximum flow rates we have observed in common residential size meters. The clock speed for the Inter-Integrated Circuit (I2C) controls the data transfer between the sensor and the datalogger and supports standard and fast sampling modes at 100 and 400 KHz, respectively [33]. Multiple data transfers from the sensor to the datalogger were measured. Each transfer takes less than 0.7 ms; therefore, conducting 570 transfers in a second would take less than 0.4 s. Based on this data, we adopted the standard configuration after observing that it is fast enough to handle all data transfers between the sensor and the datalogger.

Although it is acknowledged that the relatively weak magnetic signal produced by the water meters we tested occupies a small portion of the sensor’s potential output range (i.e., a linearly scalable integer between −128 and 127 at the ±4 gauss range), we were unable to find an inexpensive sensor with a range more suitable than the LIS3MDL. Additionally, the LIS3MDL draws less current than many other types of sensors (e.g., Hall Effect sensors) that do not provide a better range. Using an analog sensor would have required additional signal processing components and the use of the Arduino’s onboard Analog-to-Digital Converter (ADC), which would have increased power consumption and reduced the autonomy of the datalogger. At a cost of less than $5 USD, the LIS3MDL magnetometer was the best and most practical sensor we could find for this application that worked well when paired with the filtering procedure described above.

#### 2.3.2. Microcontroller Board

**Figure 4:**
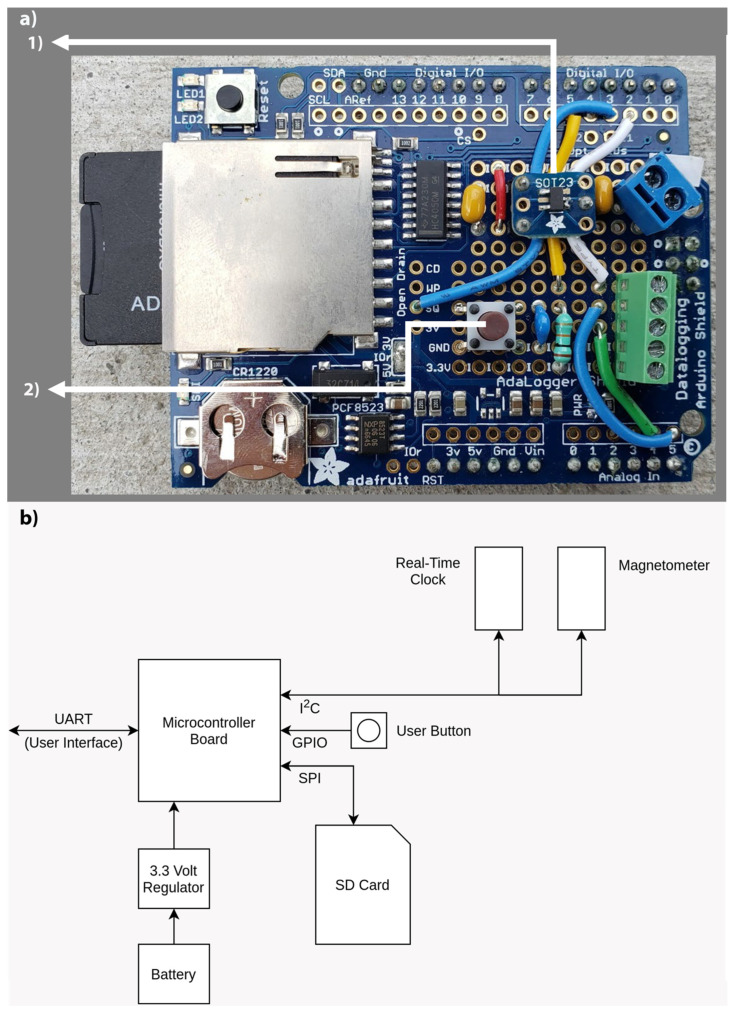
(**a**) Datalogging shield modified for the CIWS datalogger. Main external components added include: (1) more efficient power regulator installed on the Adafruit SOT23 Breakout Pack and (2) wake power button installed to access the user interface when desired. Other modifications and components can be observed in this figure, including the SD card, pin connections described, terminal blocks, resistors, and capacitors added to the shield. (**b**) Block diagram of the connections between main components in the CIWS datalogger. A full diagram of connections is available on the project’s GitHub.

The Arduino Pro is a microcontroller board based on the ATmega328 processor [34]. We chose it for this application because it is inexpensive, the absence of connectors and additional hardware components make it more customizable, the pin layout is compatible with Arduino Shields, and it is openly available. The Arduino Pro used in this system is the 3.3 V/8 MHz version. We made several modifications to minimize power consumption of our datalogger. Although the Arduino Pro has an integrated power regulator, we removed it and replaced it with a more efficient 3.3 V regulator installed on the data logging shield (Figure 4a.1). The power LED on the Arduino Pro was also removed from the board. The ATmega328 has the following peripherals: I2C, Timer 0, Timer 1, Timer 2, Serial Peripheral Interface (SPI), Universal Synchronous-Asynchronous Receive-Transmit (USART), and an Analog-to-Digital Converter (ADC) which are, by default, clocked by the microcontroller’s system clock, causing them to consume power while not in use. The Arduino manufacturer added an eight-bit memory-mapped Power Reduction Register (PRR). The bits written to this register either activate or shutdown the clock signal to a specific peripheral. In our device, all of the peripherals mentioned are turned off using this register to reduce energy consumption. The ADC and all of its timers remain off for the entire operation of the device, while the firmware developed activates the SPI, the USART, and the I2C modules when needed and turns them back off when they are no longer in use.

#### 2.3.3. Data Logging Shield

Adafruit’s data logging shield was originally designed to work with the Arduino Uno. We adapted it to work with the Arduino Pro, which has fewer connections and is more compact than the Arduino Uno. As purchased, the shield integrates a RTC for precise timing and an SD card memory slot for storage of observed data. However, several hardware modifications were needed on the logging shield to make it compatible with the Arduino Pro. First, we shorted the Serial Clock (SCL) and the Serial Data (SDA) jumpers on the bottom of the shield with solder, connecting the I2C bus to the Arduino Pro’s I2C pins. Second, we shorted the input/output resistors (IOr), 3 V, and 5 V busses together. This connects the I2C pull-up resistors to the 3.3 V bus. Since the Arduino Pro selected for the system is the 3.3 V version, this means that the pads listed as 5 V are converted to 3.3 V connections. Third, we removed the power LED from the logging shield, which reduces energy consumption. The voltage regulators on the logging shield were also removed as they are unnecessary given that a more efficient regulator was installed. A wake button was installed in the logging shield to provide a way for the operator to access the user interface designed to interact with the device (Figure 4a.2).

Figure 4b shows the diagram of the connections between the main components of the CIWS datalogger. The real-time clock (RTC) on the data logging shield keeps track of the current time. Every time step it generates a pulse on the shield’s SQ pin, which is wired to the D3 pin on the Arduino Pro (visible in Figure 4). The RTC shares the I2C bus on the Arduino Pro with the magnetometer. When the Arduino receives the pulse at the selected time step (which can be modified to meet different research needs) from the RTC, it gathers date and time information from the RTC via the I2C bus and the number of pulses detected for the current time step and logs both in a CSV file stored on the SD card. During laboratory tests and deployments, 8 and 16 GB SD cards have been used interchangeably.

#### 2.3.4. Deployment Hardware

**Figure 5:**
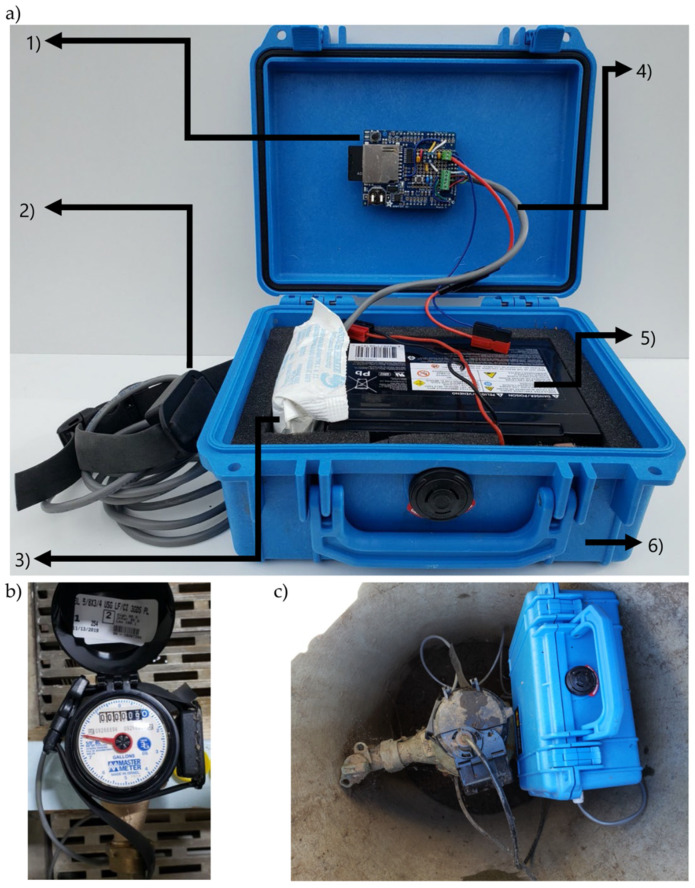
Assembled device ready for deployment. (**a**) Main components: (1) Arduino Pro and Datalogging shield coupled together, (2) potted and encapsulated LIS3MDL magnetometer sensor, (3) desiccant pack, (4) cable connecting the magnetometer and the shield, (5) 12 V 10 Ah battery, and (6) waterproof box. (**b**) Example of the sensor configuration when it is installed on a 5/8 in Master Meter meter (the orientation of the sensor does not affect the functioning of the device). (**c**) Deployment of a CIWS datalogger on a meter pit (on a 1 in Neptune T-10 meter).

For deployment, the sensor is wired to the screw terminals on the data logging shield, which is plugged in on top of the Arduino Pro (Figure 5a). The main connections between all the components of the system are represented in Figure 5, which is included to illustrate all the hardware elements. The system can be powered by any battery with a voltage equal or larger than 4 V, although power consumption will be most efficient with 12 V batteries. During testing and field deployment, a 12 V 10 Ah lead acid battery was used. Once built, the datalogger is encased in a waterproof box (Figure 5a). The magnetometer is attached to the meter’s register by using a strap. The magnetometer can be installed in any place on the outside of the register, after which logging is started and the datalogger begins collecting data, as observed in Figure 5c.

Table 1 lists all components, source, and approximate cost per unit, at the time of this writing, to build a CIWS datalogger. According to Table 1, the approximate cost to build a CWIS datalogger is around $150, which will slightly vary depending on the number of loggers built. Some parts, including cables and connectors, are only available in quantities larger than what is needed for a single datalogger. The costs presented in Table 1 were estimated after purchasing the materials to build 20 CIWS dataloggers. Part numbers and a specific link to each vendor are available in the project’s GitHub repository.

#### 2.3.5. Printed Circuit Board Design

**Figure 6:**
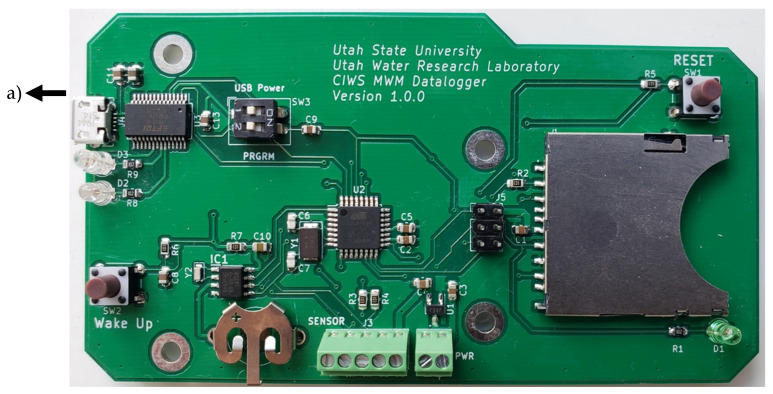
CIWS datalogger built using the printed circuit board (PCB) design developed. (a) Micro-USB connector. Other important components of the datalogger are also observed: the wake up and reset buttons, the sensor and power connections are easily identifiable in the figure by reading the inscriptions included. The SD card adapter, the coin cell battery holder, and LED light are also visible.

As a final step in realizing our hardware design, we translated our prototype datalogger into a PCB design that can be used to reduce the time and effort required to manufacture the CIWS datalogger. This PCB design includes all of the Arduino and datalogging shield components and simply needs to be connected to the LIS3MDL magnetometer sensor and the power source. We ordered a small run of five of these devices using our design from the PCBWay PCB manufacturing company (http://pcbway.com) and successfully tested them in the laboratory using the same procedures we used to test our prototypes (described below) to verify the correct functioning of these devices. The total cost for manufacturing and assembling a device (Figure 6) was $90 USD, which included manufacturing of the PCB and placing of all of the components to create a finished product. This cost can be reduced if a larger number of devices is ordered. All of the information needed to manufacture this PCB design, including schematics showing how all the parts are connected; Gerber files containing configuration parameters, aperture definitions, and coordinate information for the location of parts; and a list of the materials required is publicly available in the project’s GitHub repository. To connect a computer with this version of the CIWS datalogger, a micro-USB cable is used (Figure 6a) highlights this connector).

### 2.4. Firmware

The firmware for the CIWS datalogger is organized using a traditional, C-like Arduino programming approach [37] and is developed within the Arduino IDE, which is open source and freely available for Windows, Mac, and Linux operating systems [19]. Traditionally, C/C++ code is separated into a declaration or header (.h) file and implementation or source (.cpp) file [37] that, when precompiled together, are known as a library [38]. For the CIWS datalogger, multiple libraries were developed. For each of these libraries, the header and implementation files are available in the project GitHub repository, along with documentation about the functions developed within each library, including their output types, variables created, and data formats. Table 2 lists the library names and their main functions. In addition to the libraries listed on Table 2, other existing Arduino libraries were used in the firmware, including the serial peripheral interface (SPI) library [39], the SD library [40], the Wire library [41], and multiple “AVR Libc” libraries [42].

The main datalogger firmware file, “Firmware.ino,” calls all of the libraries mentioned to operate and control the CIWS datalogger. It is the starting point of the firmware and contains six functions: (1) *setup()*, (2) *loop()*, (3) *INT0_ISR()*, (4) *INT1_ISR()*, (5) *storeNewRecord()*, and (6) *bcdtobin()*. The *setup()* function is called once when the device is powered, and the *loop()* function runs continuously as long as the microcontroller is powered. The functions *INT0_ISR()* and *INT1_ISR()* are both interrupt service routines. An interrupt service routine is executed when an event in hardware occurs. The main *loop()* function checks these flags, and if they are set, responds accordingly. This is good practice as interrupt service routines need to be kept as short as possible [38]. Table 3 lists the main objective of these 6 functions that comprise the firmware of the CIWS datalogger and are included in the Firmware.ino file.

### 2.5. User Interface

We developed an interactive user interface within the datalogger’s firmware code that allows users to execute basic functions needed to configure and operate the datalogger along with managing and retrieving logged data files. Through this interface, the datalogger can be configured to work with different meter brands and sizes, and users can also create simple deployment information like a site identifier that makes it easier to identify and manage datasets after they have been collected. The principle of functioning, threshold values used in the Schmitt Trigger function, and data transmission rates remain constant regardless of the brand and size of the meter selected. However, the data recording frequency and the meter’s pulse resolution can be stored in the datalogger’s memory to accurately specify the volume of water associated with every observed peak (or “pulse”) in the meter’s magnetic field. Configuring the pulse resolution for a specific meter allows the user to observe volumes of water registered without interrupting data collection, which is useful in verifying correct deployment of the sensor.

The user interface can be accessed through any serial console emulator or using the Arduino IDE. After connecting a computer to the datalogger using a USB Transistor–Transistor Logic (TTL) serial cable or a USB cable with a Future Technology Devices International (FTDI) breakout module (in the case of the prototype datalogger—a standard USB micro cable is used for the PCB version), clicking the Wake button on the datalogger shield will allow access to the interactive user interface in the serial console. The message “Logger: ready” will be displayed on the screen, and the list of commands in Table 4 will be accessible. For actions with multiple options, an interactive menu will be displayed allowing users to choose the desired action/configuration.

## 3. Calibration and Implementation

The volume of water that passes through the meter per pulse measured by the datalogger, referred to in this document as the *pulse* *resolution* of the meter, must be defined in order to obtain an accurate estimation of water usage. Meter manufacturers generally do not publish this information. We determined the pulse resolution for a number of different brands and sizes of commonly used residential meters using an experimental testing facility in a laboratory setting. We chose meters used extensively by municipalities in our surrounding area (e.g., Logan City and Providence City, UT), although our testing and calibration methods could be applied to any magnetically driven water meter. Laboratory experiments were conducted with our datalogger to ensure that it can accurately measure water use at the different flow rates commonly experienced with residential water use. In these experiments, water was passed through the meters at multiple flow rates ranging from 4.43 to 86.78 LPM. The register for each meter was manually read before and after each run, allowing the volume of water used in each run to be determined by the difference in manual meter readings. The volume registered by the meter was then divided by the total number of pulses observed by the CIWS datalogger during the experimental run to calculate the meter pulse resolution, R (Equation (3)):

(3) $$R=\frac{V_{m}}{P}$$

where *V_m_* = the volume of water that passed through the meter (liters) and *P* = the number of pulses observed by the datalogger.

This process was repeated multiple times at increasing flow rates, each of which resulted in an estimate of the meter’s pulse resolution. We also verified our results using more than one meter of the same size and brand plumbed in series with separate dataloggers on each one. Table 5 presents the results of one of the calibration experiments conducted, where six runs at different flowrates and durations were observed. In this experiment, two Neptune T-10 meters of 1 in size were installed in series on the same pipe, measuring the same flow. During each run, manual meter readings from both Neptune T-10s (named “M1” and “M2” in Table 5) were taken before and after running water and were used to calculate the volume of water that passed through each meter. A CIWS datalogger was installed on each of these meters. DL1 was installed on M1, and DL2 was installed on M2. The pulses counted by each of these dataloggers were logged for each of the six runs conducted. The volumes read manually on the meters and the pulses observed by the dataloggers were then used to calculate the pulse resolution of each meter. Continued experiments demonstrated that the pulse resolution of each meter is consistent across meters of the same size and brand and across flow rates, which can be also observed in Table 5 by comparing the calculated pulse resolution of each meter.

Using this procedure, we determined that the pulse resolution for a 1 in Neptune T-10 meter is 0.1257 L/pulse, which we calculated as the average of the pulse resolution values for both meters across the six runs conducted. The standard deviation of this value was 8.757 × 10^−5^, and the coefficient of variation was 0.26%. These values demonstrate that while there is some variability in the pulse resolution values across the meters and runs, it is small enough that the calculated pulse resolution value can be used across meters of the same model/size and across flowrates.

Similar experiments were conducted using 5/8 in Neptune T-10 meters and 1 in and 5/8 in Bottom Load (BL) Master Meter meters. Table 6 lists the calibrated pulse resolution values for all of the meters we tested. These pulse resolution values were used in all field deployments of the datalogger. To calculate the volume observed by a CIWS datalogger installed on a meter, the number of pulses recorded by the datalogger is multiplied by its corresponding pulse resolution value. Introducing the meter pulse resolution in the datalogger’s user interface allows the user to visualize the volume that CIWS datalogger has registered since logging started in units of gallons (to match the register’s units). This function is useful when deploying datalogger for the first time in a meter, to ensure the installation was successful. As mentioned in Section 2.2, the output file includes the number of pulses and not volume.

**Figure 7:**
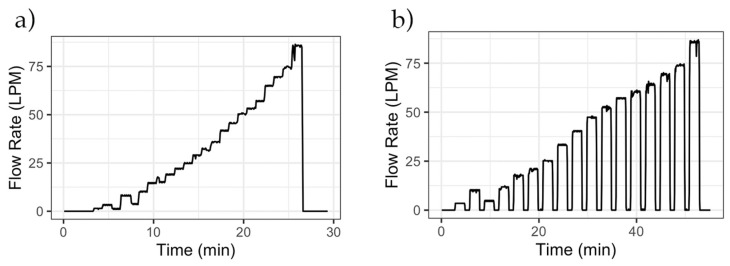
Flow signatures of the experiments used to verify the functioning of the CIWS datalogger: (**a**) increasing the flow rate gradually and (**b**) increasing the flow rate with intervals of no flow. Data shown are for a 5/8 in Neptune T-10 meter.

After the pulse resolution for each meter was calculated, the dataloggers were further tested for accuracy under different flow scenarios, e.g., for capturing rapidly changing flow rates and events of short duration, as these are common situations in residential settings. We conducted two additional laboratory experiments. In Experiment 1 (Figure 7a), the flow rate through the meter was varied by opening a flow-controlling valve. The flow rate through the meter was increased in steps without interrupting the flow between flow rate increases. We then conducted a separate experiment (Experiment 2, Figure 7b) where the flow rate through the meter was increased in steps, but the flow-controlling valve was quickly closed between each flow rate change. Manual readings of the meter’s register were taken before, during, and after each experiment to compare the volume registered by the meter’s register with the volume registered by the datalogger. The flow rate signature of Experiment 2 is similar to the signature of the experiments conducted to calibrate the device (Table 5).

In both experiments presented in Figure 7, a CIWS datalogger was installed on top of a 1 in meter and another on a 5/8 in meter. The registers for both meters were read at the beginning and end of each experiment. The volume registered by the meter and the CIWS datalogger were 777.86 and 779.33 L, respectively, with a percent error of 0.19%. For the 1 in meter, the volumes were 782.48 and 779.99 L for the meter and the CIWS datalogger, respectively, with a percent error of −0.57%. The difference in volume recorded by the 5/8 in (777.86 L) versus the 1 in (782.48) meters is not a subject of this investigation as the percent errors for the CIWS datalogger were calculated relative to the meter on which they were installed. Our goal was to ensure that the datalogger accurately reflects the corresponding meter reading. If the accuracy of the meter itself is compromised, so will be the accuracy of the measurements made by our datalogger. In Experiment 2, meters were read after each incremental increase in flow. Table 7 shows the volumes read by each meter and its corresponding CIWS datalogger along with the percent error for each step. The maximum error observed in this experiment was −0.66% for the 1 in meter and 0.61% for the 5/8 in meter. Multiple experiments of the same kind were conducted, and the error was less than 1.5% in all cases, with values being similar to those presented in Table 7.

## 4. Field Deployments

In addition to our laboratory testing, the CIWS datalogger was installed on the water meter at 5 houses in the cities of Logan and Providence, Utah, between May and September 2019 to evaluate its performance under field conditions. Table 8 shows the dates the datalogger was installed on each site. The evaluation of the CIWS datalogger performance presented in this section is based on battery life, accuracy of the measurements, errors, and limitations observed in the field. Analysis of the data collected and potential products that can be derived from it are included to illustrate potential applications of the CIWS datalogger. Data was collected using a temporal resolution of 4 s to allow the identification and posterior classification of events of short duration. The anonymized datasets and the code used for the computations presented in this section are publicly available [43].

### 4.1. Battery Life

In each deployment, the voltage was measured before and during data collection. A 12 V, 10 Ah battery was used in each deployment. Batteries were fully charged before deployment, up to 13 V and were replaced at approximately 20% of charge (~11.58 V). From fully charged to 20% of charge, at the 5 sites installed, the average discharge time was between 5 and 6 weeks indicating that the devices could reasonably be used to collect a month of 4 s temporal resolution data before the batteries have to be replaced.

### 4.2. Limitations and Errors

An obvious limitation for the installation and operation of the CIWS datalogger is related to the accessibility of the water meter. In areas around Logan, UT, meters are installed underground within a covered meter pit to ensure that they do not freeze during the winter. We encountered meter pits of depths ranging from 20 to 80 cm during field deployments, see Figure 5c for a reference. The depth of the meter in the pit affects its accessibility. In cases where the meter is within the reach of the person installing the magnetometer sensor, the process is straightforward and can be successfully completed by the installer in a few minutes. In cases where the meter is deep enough that it is not within easy reach of the installer, installation requires tools to extend the installer’s reach. In our field experiments, we found that ensuring proper placement of the magnetometer sensor and the proper functioning of the CIWS datalogger required some trial and error for meters that could not be easily reached. In this scenario, multiple visits to a same location and constant supervision of the data collected were required to ensure the accuracy of collected data. Once the datalogger was installed and functioning properly on top of a water meter, few data collection problems were observed. Early in our field trials, several of the magnetometer sensors failed, presumably because of the humidity in the meter pit. These failures caused the dataloggers to stop working and created errors in the data collected. We were able to fix this problem by covering the sensor and all the wiring connections to it with potting material. After this modification was done, we did not observe additional sensor failures. The Datalogging Shield and the Arduino Pro were protected from humidity inside the waterproof box, in which a desiccant pack was added for extra protection from humidity. Two meter pits were completely flooded during the data collection period. In one case, the magnetometer failed after the flood. In the other, the device continued to work after the pit was dry. In both cases, the datalogging components were kept relatively dry inside the box and continued to work after they were dried. No other environmental factor has been identified to affect the measuring process or damage the CIWS datalogger.

Another error observed during the field deployments was related to writing data to the CSV file. Some files became corrupt, and significant data loss occurred. We were unable to trace the origin of this error completely, although memory-related errors on the Arduino or power failures were identified as possible causes. In an effort to diagnose this error, a test was conducted by logging data over an extended period of time on multiple devices in the laboratory. Memory and battery on the device were tracked and logged into a CSV file during these experiments using the SD Arduino library [40]. Memory errors were ruled out as the cause because we observed that memory handling was effective. Power failures while writing data to the SD card using Arduino-based devices have been identified by other authors to cause data loss [26]. This cannot be discarded as the potential cause of the errors, but we were unable to diagnose them because we did not observe any issues during our laboratory testing. Although we were unable to fully diagnose these errors, the data loss problem was corrected by introducing an update into the datalogger firmware that checks to see if the data saved to the CSV file has errors. If errors are found, the CSV file is ended and a new one is automatically initiated. This firmware modification was introduced close to the end of the field data collection period, and the error was not observed again after it was implemented. As an additional safety measure to limit potential data loss in case of reappearance of the error, the firmware was modified so that a new CSV file is started every day, whereas in the original firmware a single CSV file was used for data collection periods of any length.

The Datalogging Shield has a SD card memory slot, which can fit SD/MMC storage within a range of 32 MB to 32 GB [35]; in this application, only SD cards were used. A week of data collected with a 4 s recording interval is approximately 5 MB in size. Thus, data storage does not constitute a significant limitation for the system’s autonomy. In the field campaign conducted to test the device, old and new CSV files were kept on the 16 GB SD cards for redundancy purposes. A 4 GB SD card is sufficient to handle multiple years of data, even if a smaller time step for data collection is selected.

### 4.3. Accuracy

We performed our calibrations using newly purchased meters. Although we acknowledge that the performance and accuracy of the meter itself may change over time [44,45], given that the meter’s register and our datalogger use the same spinning magnet to quantify flow through the meter, volume observed by the datalogger will match the volume recorded by the meter’s register regardless of the meter’s age. The meters observed during the field deployment were of different brands, types, and ages. Since the CIWS datalogger does not directly measure flow through the meter, it can only accurately count the magnetic pulses from the meter. Thus, the accuracy values reported in this section assume that water use calculated by subtracting manual readings of the meter’s register reflect the true value. The meter’s register at each site was read periodically to allow comparison between the volume registered by the meter and the totalized volume observed by the CIWS datalogger.

**Figure 8:**
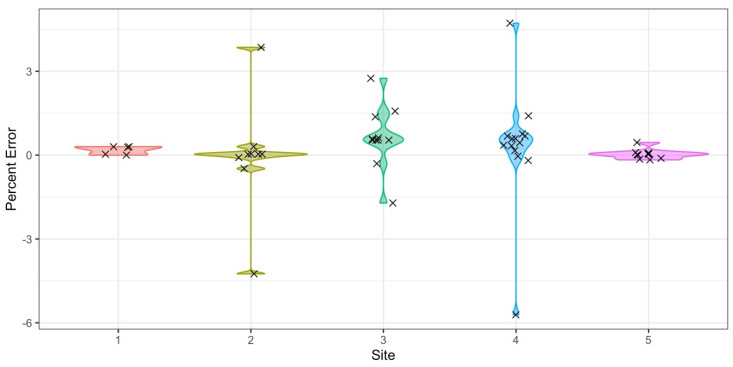
Percent difference between the volume registered by the meter (calculated as the difference between two consecutive, manual readings of the meter’s register) and the volume registered by the CIWS datalogger (calculated as the number of observed pulses multiplied by the pulse resolution of the meter) for multiple deployment periods at each experimental site. The points in the figure represent individual percent errors computed for every field visit at each site. Values have been spread across the x axis for visualization purposes. The number of deployment periods was 5, 9, 11, 14, and 10 for each site.

In the initial phase of the field deployment process, the accuracy observed was lower due to inexperience reading water meters and difficulties in the sensor installation process that were previously discussed. Figure 8 shows the percent difference between the volume registered by the meter, calculated as the difference between two consecutive readings of the meter’s register, and the volume registered by the CIWS datalogger, calculated as the total number of recorded pulses multiplied by the pulse resolution of the meter. All points calculated are presented in Figure 8 using a violin plot to present the distribution of the error values we observed in the field with the CIWS datalogger.

During laboratory experiments, volume calculations using the CIWS datalogger were all within ±1.5% of the meter volumes. In laboratory conditions, we had easy access to the meter, and volumes were calculated over relatively short periods of time when compared to the field deployments. The values observed in Figure 8 for field deployments range between ±5%, although most are within the ±1.5% range, similar to what we observed in laboratory experiments. Most values outside this range were caused by errors or sensor installation problems. Sites 2, 3, and 4 were the first three sites installed in the deployment process and served as experimental sites. At Site 4, the meter is beyond the reach of the installer, which represented a problem during the installation process. When there is water use occurring in the home at the same time logging is started or stopped, small differences between the manual meter readings and the datalogger totals can be introduced given that it is hard to read the meter’s register when it is moving. As the installers became more experienced, most problems were addressed, evidenced by the significantly smaller errors for Sites 1 and 5, which were installed on a later date than the other 3 (see Table 8 for specific dates). Deployment periods where the CSV file became corrupt on the SD card are not included in Figure 8 as the water usage data in these files were not reliable.

### 4.4. Water Use

A data recording interval of seconds, rather than minutes or longer, enables the use of end use disaggregation algorithms [46], limits the volume of leaked water that can go undetected, and decreases the error in the estimation of peak demand [14]. Disaggregation of end uses is a complex process, particularly for overlapping events [10,47,48,49]. The purpose of the analysis presented in this section is not to produce a disaggregation/classification algorithm but rather to demonstrate the potential for using data collected using the CIWS datalogger as input to such algorithms for disaggregation and classification of water end uses. The first step in this type of analysis is to identify water use events, followed by disaggregation of simultaneous or overlapping events, and finally classification of individual events by type. For simplification, water use events in this analysis were identified as periods of nonzero flow—an event starts when the pulse count is larger than zero and ends when the pulse count is zero again. This simplified approach for separating events may lead to uncertain results when there are continuous leaks where the pulse count does not return to zero between events. It also does not consider overlapping events.

**Figure 9:**
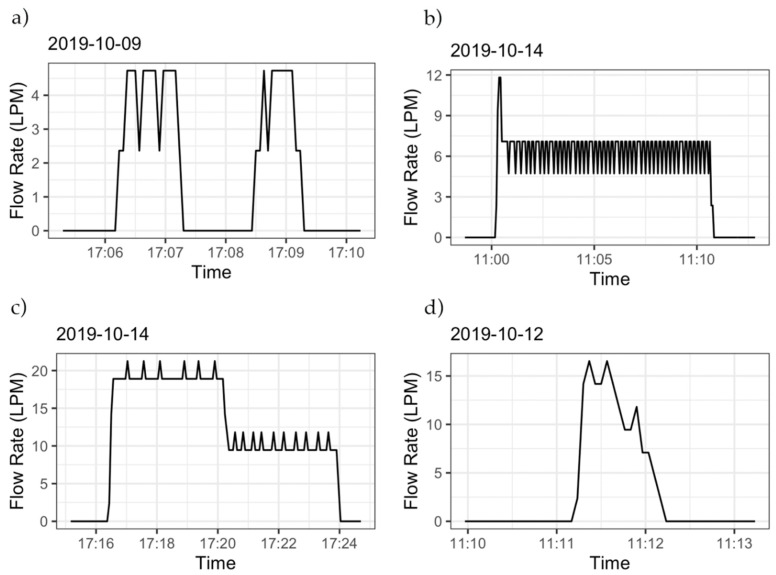
Flow rate signatures for events labeled by the homeowner at Site 1. Panel (**a**) two subsequent faucet events, (**b**) a shower event, (**c**) a clothes washer event, and (**d**) a toilet flush event.

Using this simplified approach, we identified 5838 events at Site 1, 2133 at Site 2, 73,975 at Site 3, 2647 at Site 4, and 3777 at Site 5. In order to identify and label some of these events, the homeowner at Site 1 was asked to log the start time and type of water use events in their home. Table 9 lists a sample of the events logged by the homeowner, and Figure 9 shows the data for the date and time of these events. Figure 9a shows two subsequent faucet events. Flow rates in these events are similar, but duration is different. Figure 9b–d represents a shower, clothes washer, and toilet flush event, respectively. The flow rate and duration of water use events depend on the characteristics and setting of the fixtures and on personal preferences of the user. The oscillations between flow rates within each of the events are related to the data recording interval and the pulse resolution of the meter. Since only discrete pulses can be counted when flow rates are relatively constant (e.g., within an event), the pulse counts within adjacent recording intervals may vary by ±1 pulse, leading to the flow rate behavior shown in Figure 9. The homeowner at Site 1 labeled multiple events; however, not all the events of the same kind exhibit the same pattern in terms of flow rate or duration. Duration, volume, and flow rate have been used to identify end uses of water by finding similarities among events using multiple methodologies, ranging from visual identification to machine learning algorithms [47,48,50].

**Figure 10:**
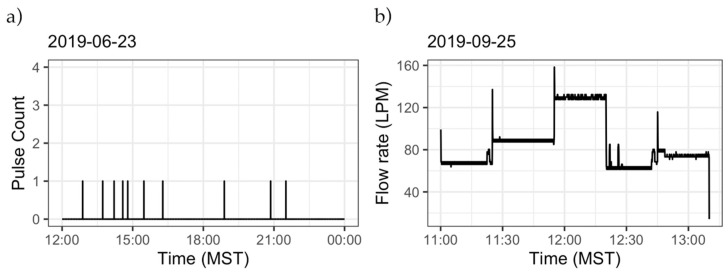
Sample of the events observed. (**a**) Raw data collected at Site 5 on June 23, 2019 from noon to midnight. Multiple 4 s, single pulse events were observed at a time when no other water use occurs. (**b**) An irrigation event at Site 1. At this temporal resolution, flow rates from different irrigation zones can be observed.

Some events have characteristics that make their identification easier than others. Events with a duration of 4 s (the temporal resolution of the data collection) or less and only one pulse are likely to be leaks. Events with duration and/or flow rates much larger than most events at a site are likely to be outdoor irrigation events. Figure 10a shows leaks occurring at Site 5 in a period of approximately 12 h when no other water use occurs. If all of the events lasting 4 s (or less) are assumed to be leaks, we can calculate the leak rate, resulting in leakage of 6.7 L/d at Site 1, 2.2 L/d at Site 2, 48.4 L/d at Site 3, 0.5 L/d at Site 4, and 7.2 L/d at Site 5. Other studies have found that leaks represent, on average, 13% of the indoor water use and average 64.3 L/d [6]. However, their definition of leaks includes more events than those described here, and indoor water use is not fully assessed in this analysis. Figure 10b presents an irrigation event (identified by its long duration and large volume) at Site 1. Irrigation events will exhibit a different pattern depending on whether a manual or automated system is used, the number of “zones” that are irrigated, and the number and type of sprinkler heads within each “zone.” At Site 1, for the event presented, an automated sprinkler irrigation system is used with five different irrigation zones. We also observed overlapping events occurring (between 12:15 p.m. and 12:30 p.m.).

**Figure 11:**
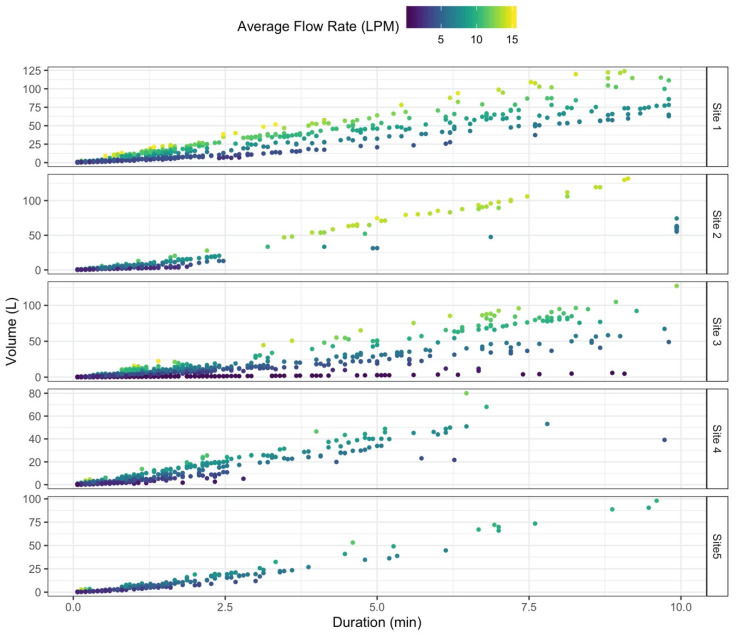
Duration (minutes) versus volume (liters) of 23,478 events logged at the 5 sites. Events presented are those with a duration of less than 10 min and average flow rate less than 15 LPM (96% of all the events that are not leaks). Color is assigned to each event based on its average flow rate (LPM). These three values have been used by other authors to identify and classify end uses events.

From the total number of events identified using our simplified procedure, 39% (Site 1), 48% (Site 2), 91% (Site 3), 18% (Site 4), and 89% (Site 5) were classified as leaks, resulting in 85% of the total combined events being leaks. Of the remaining 15%, approximately only 1.3% had a duration larger than 25 min, which are likely to be irrigation or overlapping events. None of the participants reported having a swimming pool. Most (96%) of the non-leak events had a duration less than 10 min and an average flow rate less than 15 LPM. Figure 11 presents the duration and volume for these events at each site. Volume and duration alone do not seem to discriminate different types of events. However, when adding the average flow rate (colors of the points), levels between the events begin to appear. Events at these shorter durations should include toilets, which have similar volumes, durations, and flow rates along with faucets and showers, which will have different duration and volumes but occur at similar flow rates. Dishwasher and clothes washer events should also be similar, although varying designs, manufacturers, and available cycles would contribute to differences. The distribution of events also varied among the sites, which could indicate differences in personal preferences, water fixtures, or both. Although a rigorous clustering analysis is beyond the scope of this paper, Figure 11 shows that even 3 calculated event attributes begin to illustrate differences in event types. All of the event statistics, including those not shown in the figure (e.g., mode flow rate and maximum flow rate), could be used as factors in a more sophisticated clustering approach to classify each event into end use categories.

The high temporal resolution data allows for calculation of other important characteristics of water use, such as instantaneous peak, hour peak, daily average, and daily maximum water use. Table 10 shows these statistics for each of the sites. Data collection periods are not concurrent for all the sites, which could explain some of the difference observed in the per capita daily average. Utah daily average water use is approximately 632 L per capita [51]. Water usage has a large seasonal component corresponding to landscape irrigation, and the data collection period from this experiment is not long enough to capture the annual variability. The majority of sites exhibited high variability among daily, hourly, and sub-hour resolution, adding supporting evidence to the claim that there are water use patterns masked in coarser temporal resolution data, such as hourly, daily, or monthly values. Values such as the peak hour maximum daily use are typically estimated from coarser temporal resolution data or calculated based on typical characteristics of a household, which add significant uncertainty to the management and design of water networks [52].

## 5. Discussion and Conclusions

A low-cost, open-source datalogger for collecting high temporal resolution water use data has been presented. The system can be installed on top of existing, analog, magnetically driven water meters without affecting their functionality. The hardware components we used to prototype the datalogger are readily available, and assembly is straightforward using supporting materials provided. For potential users who do not want to assemble dataloggers from components, we have provided a PCB design that can be used for commercial manufacture and assembly. All of these materials, along with the code of the open-source firmware developed for operating the datalogger are open source and available, making it possible for any researcher to use our datalogger design as presented here or to modify our design to develop their own systems. The CIWS datalogger can potentially work with a wide range of magnetically driven, positive displacement meters existing worldwide, although validation and calibration of the datalogger with each meter type and size is required before extending its application beyond the specific water meters we tested. The logger can be configured to collect data at any temporal resolution required, which represents an improvement over other existing commercial products. The advantages of using the CIWS datalogger are that its cost is significantly lower than other existing technologies for collecting high temporal resolution water use data (~$150 USD versus more than $2000 USD), it does not disrupt the functioning of the meter on which it is installed, and it does not require plumbing or disruptive installation. Although we performed our calibrations in a controlled laboratory setting, calibration for other meter types could be achieved by following the methodology presented in this paper with the datalogger installed on meters deployed in the field.

Battery life constitutes the biggest limitation in terms of autonomy of the CIWS datalogger. Using a 12 V, 10 Ah battery, we were able to get between 5 and 6 weeks of autonomous operation with a data recording interval of 4 s. However, this battery life has been sufficient for our data collection needs and exceeds the period for which proprietary dataloggers [8] used in past studies can be deployed to collect data at similar temporal resolution (i.e., 7.5 days of data with 5 s recording interval). Adding a solar panel or an additional power source can extend the autonomy of the logger. Our results indicate that there is a learning curve for reading existing meters and for developing the skills needed to properly install the sensor. Accuracy increases once this period of learning has elapsed. The differences in the volumes observed by the CIWS datalogger and the meter’s register indicate that the system presented is accurate within approximately 2% of the meter readings, when properly installed on the Neptune T-10 and Master Meter BL meters we tested under field conditions. For new installers, or when the meter pit is deep, this value can be as large as 5% of the meter reading. We were unable to find accuracy information for proprietary Meter-Master 100 Flow Recorder devices used in past studies [6], although their documentation reports that accuracy is assessed by comparing the volume recorded by their datalogger to the volume recorded by the meter’s register as we did in all of our tests. We did not have access to test one of these devices in the laboratory for comparison. The accuracy of the CIWS datalogger we observed in our laboratory testing was very similar to accuracy values reported for the proprietary Flume device [15] (i.e., within 1−2% depending on flow rate), which is the only other similar device of which we are aware. The CIWS datalogger should work with any magnetically driven meter, which is not the case for the Flume device, although further testing and calibration of the pulse resolution parameters for other meter brands and sizes is recommended before installing the CIWS datalogger on meters outside the ones presented here.

For simplicity, and given the small size of our field deployment, we used the original CSV files recorded by the dataloggers to obtain the results presented. The results of the field-testing campaign we conducted over 5 months indicate that the datalogger is accurate and reliable and that it can withstand the temperature and humidity conditions existing in underground meter pits during different periods of the year. The low cost (≈$150) and ease with which the datalogger can be deployed and used makes it ideal for residential water use studies that may have been cost prohibitive in the past. Results from the field campaign demonstrate that the 4 s data recording interval enables identification of daily patterns in water usage, peak timing, and volumes and accurate identification and characterization of individual end use events. Enabling disaggregation of end uses is key to fully understanding how water is used inside a monitored home and for identifying opportunities for conservation, forecasting demand, and determining how water use patterns may change over time in response to population growth, demographic shifts, and improvements in technology.

Collecting high temporal resolution data can be expensive, labor intensive, and disruptive. Newer smart meters can enable high temporal resolution data collection, but analog, positive displacement meters are still the most common meters in use within the United States. The CIWS datalogger can enable high temporal resolution water use data collection on these existing meters. The CIWS datalogger can be used by utilities for educational interventions, for assessing the outcome of conservation campaigns, for generating more accurate water demand forecasting, and for data collection in any projects that require collection of high temporal resolution water use data. Given the volume of data produced, deploying the CIWS datalogger at a wider scale will require the development of a data management system consisting of cyberinfrastructure that can enable organized transformation of the data collected into useful information. Indeed, future work will include advancing this data management cyberinfrastructure along with implementation of Wi-Fi and/or cellular communication capabilities for the CIWS datalogger, which may enable automated transmission of data from the meter into a water user’s home or to a water utility’s office.

## Figures and Tables

**Table 1:** Parts required and costs to build a Cyberinfrastructure for Intelligent Water Supply (CIWS) datalogger.

Part	Cost/Logger	Vendor
3.3 V 8 MHz Arduino Pro (ATmega328p board)	$15.95	SparkFun
Datalogging shield	$15.95	Adafruit
LIS3MDL magnetometer + breakout board	$4.95	Pololu
Male ICSP headers (2 × 15 block)	$0.65	Mouser
Adafruit SOT23 breakout pack	$0.99	Mouser
Stripboard	$1.43	Mouser
Anderson powerpole connectors	$1.30	Amazon
MCP1703 3.3 V regulator	$0.55	Mouser
Fuse	$0.17	All Electronics
In-line fuse holder	$0.45	All Electronics
Serial extender housing pack	$0.16	Pololu
Screws	$0.17	Mouser
Nuts	$0.12	Mouser
Box kit	$2.10	Mouser
Cable glands	$1.95	Mouser
Battery connectors	$1.08	Mouser
10 kOhm resistor	$0.50	Mouser
1 μF ceramic capacitors	$0.92	Mouser
0.01 μF ceramic capacitor	$0.25	Mouser
Button	$0.16	Mouser
Spacers	$0.20	Mouser
2-position terminal block	$0.90	USU ECE Store
5-position terminal block	$4.11	Mouser
Wire (battery—positive)	$0.15	Mouser
Wire (battery—negative)	$0.15	Mouser
Wire (prototype board soldering)	$0.80	Mouser
Coin cell (614-CR1220.IB)	$1.14	Mouser
Micro SD cards with adapters	$9.95	Mouser
Pelican 1150 Case (with foam)	$31.96	Amazon
12 V 10 Ah Duracell Battery	$39.99	Batteries + Bulbs
Clasp	$0.739	Amazon
5-conductor cable	$9.11	Mouser
Female headers (36 pin)	$0.59	Mouser
Strap set: Gear Strapz (+5 Clasps)	$1.52	Amazon
Total cost	$151.19	

**Table 2:** Code libraries developed for the CIWS datalogger firmware.

Library	Main Objective
state	Initializing and keeping track of: Whether the values registered have gone above or below the threshold values to trigger a pulse. The number of pulses in the current time step, time stamp, and record number. Whether the device is logging or not. Whether the serial interface is active or not. Whether the SD card has been initialized or not. Whether the magnetometer data is ready or not. Whether the configuration data is valid or not, including Site #, Datalogger ID, meter pulse resolution, and recording interval. The current filename of the CSV output file. Variables used for processing the magnetometer signal and variables used in multiple functions in other libraries.
detectPeaks	Detecting peaks in the magnetic field. Defines functions that are responsible for filtering the raw data from the magnetometer and applying the Schmitt Trigger.
magnetometer	Managing the LIS3MDL magnetometer. Defines the functions responsible for initializing and reading data from the LIS3MDL magnetometer.
RTC_PCF8523	Managing the RTC. Defines functions that are responsible for transferring data to and from the RTC, including configuration data such as the interval of data collection. Reads the date and time from the RTC that is printed in the output file.
configuration	Managing configuration data in the Electrically Erasable Programmable Read-Only Memory (EEPROM). Defines functions that: Check if the EEPROM has configuration data. Verify the correct functioning of the EEPROM. Configure the writing, reading, and the loading of data into the EEPROM data register.
powerSleep	Optimization of components for power management. Defines functions that set the Arduino Pro into standby mode, a low power consumption mode, and disable all the peripherals as described in the hardware section.
handleSerial	Operating the user interface. Define all the functions that allow the functioning and interaction with the user interface, described in the next section.

**Table 3:** Functions executed by the CIWS datalogger Firmware.ino file and main objective.

Function	Main Objective
*setup()*	Executes the following tasks: Initializes the system state data structure. Initializes General-Purpose Input Output (GPIO) pins. Initializes the magnetometer. Initializes the real-time clock. Sets up the magnetometer and real-time clock interrupt handlers. Checks that the datalogger has valid configuration data. Disables the clock for all unused Arduino peripherals. Opens the serial interface if the serial activation button is pressed.
*loop()*	The datalogger firmware’s main loop that performs the following actions: Check if the serial activation button is pressed. Run the serial menu. Check if a data recording interval has passed. Check if magnetometer data are ready. Process incoming data to count peaks. Starts a new CSV file every day while logging data.
*INT0_ISR()*	An interrupt service routine that executes when the voltage on the Arduino’s digital pin 2 transitions from low to high. The voltage signal on digital pin 2 is controlled by the magnetometer. When the magnetometer has new data ready to report, it sets the voltage on pin 2 to high causing *INT0_ISR()* to execute, indicating the main program to read data from the magnetometer sensor.
*INT1_ISR()*	An interrupt service routine that executes when the voltage on the Arduino’s digital pin 3 transitions from high to low. The voltage signal on digital pin 3 is controlled by the RTC. When the interval of time has elapsed, the RTC sets the voltage on pin 3 to low causing *INT1_ISR()* to execute, indicating the main program to read a new datetime from the RTC and store data in the SD card.
*storeNewRecord()*	Primarily responsible for storing data records to the datalogger’s SD card by performing the following actions: Gather date and time information. Activate the Serial Peripheral Interface (SPI) module’s clock. Open a CSV file on the SD Card. Print the following fields separated by commas: Timestamp, Record Number, Pulse Count. Close the file. Increment the record number. Deactivate the SPI module’s clock. Compares the number of bytes written with the number of bytes attempted and starts a new file if there are differences between these two values.
*bcdtobin()*	Responsible for converting the Binary Coded Decimal (BCD) data from the RTC into standard binary data. Takes as input a BCD value and a bitmask corresponding to the RTC register from which the BCD value came from. This conversion is accomplished by multiplying the top 4 bits by 10 and adding that number to the bottom 4 bits.

**Table 4:** List of commands, actions, and brief description of the main functions available in the CIWS datalogger.

Command	Action	Description
c	Clean the SD card	Accesses an interactive menu that allows the user to delete files from the SD card.
d	View date/time	Displays current date and time on the device.
e	Exit the serial interface	Exits the serial interface and puts the device back to sleep.
E	Eject the SD card	Allows the user to safely extract the SD card from the device for data transferring.
g	Set device configuration	Enters the configuration mode—site, file number, and meter pulse resolution are entered within this command.
h	Display help	Displays all of the configuration options available.
i	Initialize the SD card	Initializes the SD card, which must happen prior to starting to log data.
l	List all the files on the SD card	Lists all of the files currently on the SD card.
p	Print configuration data	Prints the site number, file number, and meter pulse resolution.
R	Diagnose the RTC	Checks configuration data of the RTC.
t	Change the time interval for data collection	Allows the user to set the time interval for data collection. Values can range from 1 s to more than 15 min.
s	Start datalogging	Starts logging data using the configuration data and date/time on the device.
S	Stop datalogging	Stops the data logging process.
u	Update date/time	Allows the user to change the date and time on the device.
w	Print water flow data	Displays the volume of water measured by the device since logging started.

**Table 5:** Calibration results for the CIWS datalogger using two 1 in Neptune T-10 meters.

RUN		RUN 1	RUN 2	RUN 3	RUN 4	RUN 5	RUN 6
**Duration (min)**		7	3	3	2	2	2
**Volume** **(L)**	**M1**	30.9	32.2	44.6	59.1	95.3	173.5
	**M2**	30.9	32.0	44.6	59.1	95.2	173.7
**Pulses**	**DL1**	246	256	355	470	759	1382
	**DL2**	247	255	355	470	758	1372
Average Flow Rate (LPM) *		4.4	10.7	14.9	29.5	47.6	86.8
**Pulse-Resolution (L/pulse)**	**M1**	0.125565	0.125835	0.125505	0.125643	0.125582	0.125532
	**M2**	0.125210	0.125587	0.125612	0.125643	0.125598	0.126613

* The average flow rate is calculated using the average volume between the two meters used.

**Table 6:** Pulse resolution values resulting from calibration of the CIWS datalogger for the most popular meter models in Logan and Providence Cities, Utah.

Meter Brand and Model	Size (in)	Pulse Resolution (L/pulse)
Neptune T-10	1	0.1257
	5/8	0.0329
Master Meter BL	1	0.1575
	3/4	0.0957

**Table 7:** Results from Experiment 2 for the 1 and 5/8 in Master Meter.

Time (a.m.)	1 in Meter Volumes (L)			5/8 in Meter Volumes (L)		
	Meter	Datalogger	Error	Meter	Datalogger	Error
10:15	6.78	6.79	0.14%	6.89	6.92	0.37%
10:18	20.37	20.23	−0.66%	20.21	20.28	0.35%
10:21	9.43	9.42	−0.01%	9.43	9.48	0.61%
10:24	23.09	23.12	0.13%	23.05	23.12	0.27%
10:27	35.05	35.06	0.02%	34.94	35.14	0.56%
10:30	40.58	40.71	0.33%	40.43	40.67	0.59%
10:33	50.38	50.39	0.01%	50.12	50.32	0.39%
10:36	66.43	66.47	0.06%	66.06	66.29	0.35%
10:39	80.02	79.92	−0.13%	79.49	79.49	0.00%
10:42	94.71	94.62	−0.09%	94.33	93.98	−0.37%
10:45	102.24	102.16	−0.08%	101.64	101.42	−0.21%
10:48	115.08	115.23	0.13%	114.55	114.23	−0.27%
10:51	121.97	121.26	−0.58%	120.68	120.36	−0.27%
10:54	125.11	125.79	0.54%	125.22	124.97	−0.20%
10:57	135.56	135.59	0.02%	134.95	134.71	−0.18%
11:00	149.49	149.54	0.03%	148.84	148.71	−0.09%
11:03	175.98	176.18	0.11%	175.49	175.28	−0.12%

**Table 8:** Sites where a CIWS datalogger was installed, meter characteristics, and data collection period.

Site	Start Date	End Date	Meter Brand	Size	City
1	9/20/19	10/15/19	Master Meter	1”	Providence
2	5/31/19	7/17/19	Neptune	1”	Logan
3	5/28/19	7/9/19	Neptune	5/8”	Logan
4	5/17/19	6/17/19	Neptune	5/8”	Logan
5	6/3/19	7/17/19	Neptune	1”	Logan

**Table 9:** Events logged by the homeowner at Site 1 and main characteristics calculated from the high temporal resolution data collected.

Date	Time	Type	Duration (min)	Volume (L)	Average Flow Rate (LPM)	Maximum Flow Rate (LPM)	Mode Flow Rate (LPM)
2019-10-09	17:05:18	Faucet	1.07	4.24	3.98	4.73	4.73
2019-10-09	17:07:40	Faucet	0.80	2.99	3.75	4.73	4.73
2019-10-14	10:58:19	Shower	10.60	67.76	6.40	11.81	7.08
2019-10-14	17:15:10	Clothes washer	7.60	109.02	14.35	21.27	18.89
2019-10-12	11:09:55	Toilet	1	10.41	10.41	16.54	14.20

**Table 10:** Water usage statistics calculated from the data collected.

Site	DAWU (L)	PCDU (L)	DSD (L)	DMWU (L)	MaxDU (L)	PeakHour (L)	PeakMinute (L)	PeakInt (LPM)
1	1630	326	2188	1283	12,979	5351	131	158
2	3308	1654	4048	2331	13,106	3872	134	83
3	1145	573	673	1102	2458	667	30	34
4	897	224	1316	389	5311	1718	56	64
5	14,512	7256	10,667	12,084	44,225	10,506	190	128

DAU: daily average water use. PCDU: per capita average daily use. DSD: standard deviation of daily use. DMWU: daily median water use. MaxDU: maximum daily water use. PeakHour: maximin hourly water use. PeakMinute: maximum minute water use. PeakInt: instantaneous peak, over every 4 s interval, in LPM.

## Data Availability

All of the hardware modifications, parts, PCB design, firmware code, and supplemental materials are available in the GitHub repository for the project at https://github.com/UCHIC/CIWS-MWM-Logger. A snapshot of this repository at the time of this writing was created for archival purposes and published in Zenodo [53]. The repository contains separate folders for Hardware, Firmware, and Tools. All of the firmware libraries (.h and .cpp files) and supplemental firmware documentation are available in the Firmware folder. The Hardware folder contains additional images of the logger, the hardware design, layout, PCB design, and instructions to perform the hardware modifications described in this article. The anonymized data collected at each site from our field-testing campaign and R scripts used to produce the results presented here are published in HydroShare [43]. The HydroShare resource also includes the sample of the raw data discussed in Section 3 with R scripts for its analysis along with the data from the laboratory experiments presented.
